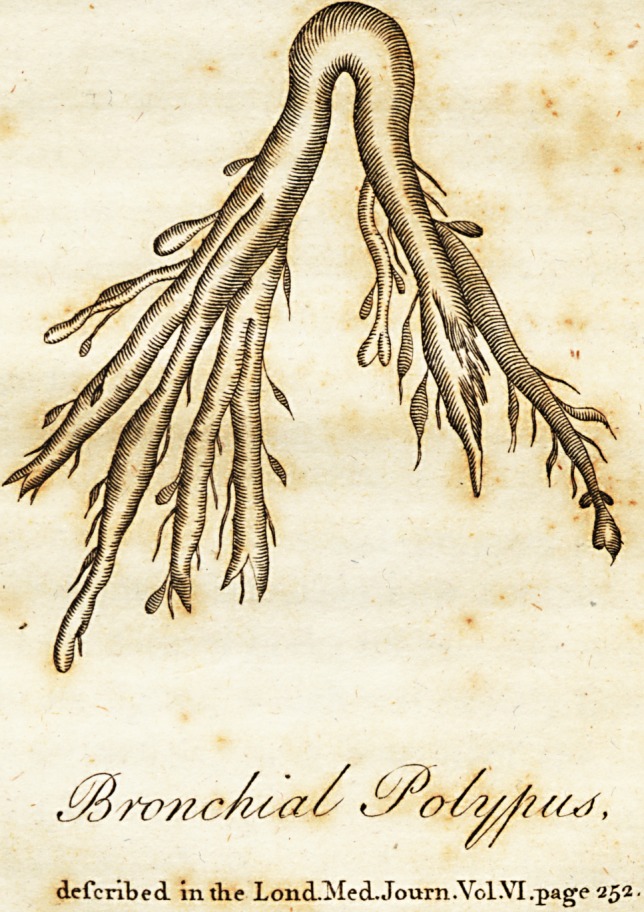# Case of a Bronchial Polypus; with Remarks on the Formation of Polypose Concretions in the Lungs

**Published:** 1785

**Authors:** 


II. *Caſe of a Bronchial Polypus; with Remarks on the Formation of polypoſe Concretions in the Lungs. By Mr.* Richard Moyle, *Surgeon at Marazion, in Cornwall.*

MR. John Pearſe, of Penzance, Surgeon, a man of a delicate conſtitution, and cloſe attention to his profeſſion, was ſeized, in the latter part of the year 1772, with an hæmoptoe, which, though ſlight, brought on complaints of the hectic kind, and returned at different times till December 9th, 1776, when the cough grew more troubleſome, and, by violent ſtraining, ruptured a large blood veſſel, ſo that a quart of freſh florid blood was thrown up, by an inceſſant cough, in a few minutes. It then abated; but the difficulty of breathing and other ſymptoms attending pulmonary conſumption, were greatly increaſed: during that day and the next, his expectoration conſiſted chiefly of coagulated blood; but on the morning of the 11th he brought up, with much difficulty, a large polypoſe concretion, of the exact

*Bronchial Polypus,*

defcribed in the Lond. Med. Journ. Vol. VI. page 252.

exact form and fize as the annexed figure: its furface was covered with a little coagulated blood; and, being thrown into water to enable us to view it more diftinctly, its finer ramifications or filaments were deftroyed by the maceration, and its furface became quite white, and of a fibrous texture, but very tender. The difficulty of breathing was greatly relieved by the removal of this concretion, and no fymptoms of a frefh hæmorrhage appeared till about midnight of the 12th, when the patient was awakened by a tickling cough, which threw up a very large coagulum of blood that ferved to plug up the ruptured veffel, and the great effufion which immediately took place in a few minutes finifhed his life.

*Remarks.*

This cafe of a bronchial polypus feems to afford us fome information how concretions of this fort are formed in the lungs. We will firft confider its figure, which was exactly like that of the air veffels, or a branch of the afpera arteria, (fee the figure.) It confifted of two round bodies, which, joined at the upper part, making a large trunk; thefe branches, in going down, divided into feveral others, particularly larly on one ſide, each of them throwing off a ſmaller twig till they ended in very fine ramifications ſimilar to a fringe. Its colour, when firſt expectorated, was whitiſh, ſlightly intermixed with a tinge of carnation; but at this time, from being preſerved in ſpirits, it is of a dead white. Its ſubſtance, particularly at its extremities, was very ſoft and tender, ſo that ſeveral of its finer ramifications, which are not in the figure, were loſt by its maceration in water; but at preſent it is of a firm, ſolid, fleſh-like conſiſtence, compoſed of different laminæ, wrapped round each other.

The figure of this polypus, and the relief in breathing which immediately enſued on its removal from the lungs, are ſufficient proofs of its having been ſituated in the ramifications of the bronchia; and as this concretion probably took place at the time of the great hæmorrhage on the 9th of December, its formation may, perhaps, with great plauſibility, be attributed to the coagulable lymph, ſeparated from the maſs of blood, which was effuſed through the cavities of the lungs, and firmly impacted into the air veſſels by the action of reſpiration.

We are perfectly convinced, from the known properties of the component parts of the blood, that that the coagulable lymph, as ſoon as it gets out of the courſe of the circulation, always coagulates; and as conſumptive perſons are particularly ſubject to an increaſed action of the arteries, their blood, when left to coagulate, always affords more buff or coagulable lymph, and therefore, on the ſmalleſt extravaſation of blood in the lungs of ſuch ſubjects, polypoſe concretions may be liable to be formed.

The opinion here advanced differs greatly from that of Dr. Warren in the Medical Tranſactions *; but the difficulty of breathing which, in theſe caſes, comes on ſo ſuddenly, ſeems to preclude the idea that the mucous glands of the lungs, by affording a greater ſecretion, can be the ſole cauſe of bronchial polypi. — In the paper alluded to, Dr. Warren ſays †, "this is the hiſtory of the caſe from "its beginning, in February, 1764, to the "night preceding the 28th of May follow-"ing, when the quickneſs of the pulſe and "difficulty of breathing returned with as great "violence as ever: in the morning a larger "polypus was coughed up than at any time

† Ibid. page 411.

"before,

" before, and, in four days following, as great " a quantity as in the fix weeks preceding."

This information feems to ftrengthen the hypothefis here advanced, as the *fudden return of the difficulty of breathing* may, perhaps, be imputed to an effufion of fome fubftance into the lungs, rather than to the *gradual* ftuffing up of the air veffels by a regular increafed fecretion of mucus; befides, moft of the cafes of this kind have been attended with an hæmorrhage fimilar to the prefent one, particularly one mentioned by Tulpius, where the patient died from the great lofs of blood: this might, as Dr. Warren obferves, be owing to its ftrong attachments to the parts in which the polypus was formed; but, in the prefent cafe, I think it probable that the polypus ferved as a plug to fome ruptured veffel; for, on the removal of a large coagulum, the hæmorrhage, as we have feen, returned with fo much violence as to kill in a few minutes. In thofe cafes where polypi have been expectorated without any blood, it is probable that the red particles have been abforbed previous to the removal of the concretion; or fuch fubftances may have been formed from an effufion of coagulable lymph alone into the lungs.

## Figures and Tables

**Figure f1:**